# Serological Phenotyping Analysis Uncovers a Unique Metabolomic Pattern Associated With Early Onset of Type 2 Diabetes Mellitus

**DOI:** 10.3389/fmolb.2022.841209

**Published:** 2022-04-08

**Authors:** Linmin Zhu, Qianyang Huang, Xiao Li, Bo Jin, Yun Ding, C. James Chou, Kuo-Jung Su, Yani Zhang, Xingguo Chen, Kuo Yuan Hwa, Sheeno Thyparambil, Weili Liao, Zhi Han, Richard Mortensen, Yi Jin, Zhen Li, James Schilling, Zhen Li, Karl G. Sylvester, Xuguo Sun, Xuefeng B. Ling

**Affiliations:** ^1^ School of Laboratory Medicine, Tianjin Medical University, Tianjin, China; ^2^ Tianjin Teda Hospital, Tianjin, China; ^3^ mProbe Inc, Mountain View, CA, United States; ^4^ Tianjin Yunjian Medical Laboratory Institute Co., Ltd, Tianjin, China; ^5^ Binhai Industrial Technology Research Institute, Zhejiang University, Tianjin, China; ^6^ Shanghai Yunxiang Medical Technology Co., Ltd., Shanghai, China; ^7^ Department of Surgery, Stanford University, School of Medicine, Stanford, CA, United States

**Keywords:** metabolomics, type 2 diabetes mellitus, biomarker, early detection, serum

## Abstract

**Background:** Type 2 diabetes mellitus (T2DM) is a multifaceted disorder affecting epidemic proportion at global scope. Defective insulin secretion by pancreatic *β*-cells and the inability of insulin-sensitive tissues to respond effectively to insulin are the underlying biology of T2DM. However, circulating biomarkers indicative of early diabetic onset at the asymptomatic stage have not been well described. We hypothesized that global and targeted mass spectrometry (MS) based metabolomic discovery can identify novel serological metabolic biomarkers specifically associated with T2DM. We further hypothesized that these markers can have a unique pattern associated with latent or early asymptomatic stage, promising an effective liquid biopsy approach for population T2DM risk stratification and screening.

**Methods:** Four independent cohorts were assembled for the study. The T2DM cohort included sera from 25 patients with T2DM and 25 healthy individuals for the biomarker discovery and sera from 15 patients with T2DM and 15 healthy controls for the testing. The Pre-T2DM cohort included sera from 76 with prediabetes and 62 healthy controls for the model training and sera from 35 patients with prediabetes and 27 healthy controls for the model testing. Both global and targeted (amino acid, acylcarnitine, and fatty acid) approaches were used to deep phenotype the serological metabolome by high performance liquid chromatography-high resolution mass spectrometry. Different machine learning approaches (Random Forest, XGBoost, and ElasticNet) were applied to model the unique T2DM/Pre-T2DM metabolic patterns and contrasted with their effectiness to differentiate T2DM/Pre-T2DM from controls.

**Results:** The univariate analysis identified unique panel of metabolites (*n* = 22) significantly associated with T2DM. Global metabolomics and subsequent structure determination led to the identification of 8 T2DM biomarkers while targeted LCMS profiling discovered 14 T2DM biomarkers. Our panel can effectively differentiate T2DM (ROC AUC = 1.00) or Pre-T2DM (ROC AUC = 0.84) from the controls in the respective testing cohort.

**Conclusion:** Our serological metabolite panel can be utilized to identifiy asymptomatic population at risk of T2DM, which may provide utility in identifying population at risk at an early stage of diabetic development to allow for clinical intervention. This early detection would guide ehanced levels of care and accelerate development of clinical strategies to prevent T2DM.

## 1 Introduction

Type 2 diabetes mellitus (T2DM) is a metabolic disease affecting a significant population worldwide. The prevalence of T2DM is expected to expand into half a billion people by the year 2030 (Whiting et al., 2011). It is manifested as chronic hyperglycemia caused by defective insulin secretion, progressive development of insulin resistance, and an inadequate compensatory insulin secretory response (Stumvoll et al., 2005; Whiting et al., 2011). In the presence of homeostatic imbalance of glucose, the overload of advanced glycation end products induces enhanced oxidative stress and systemic inflammation, which irreversibly causes functional loss of pancreatic islet *β* cells (DeFronzo, 1988; Rossetti et al., 1990; Robertson et al., 2003), peripheral insulin-targeting tissues (Garvey et al., 1987; Rossetti et al., 1987), micro- and macro-vascular cells (Brownlee, 2001; Brownlee, 2005), leading to pathological complications such as cardiomyopathy, nephropathy, retinopathy, neuropathy, and atherosclerosis.

The early detection of T2DM remains a significant challenge in clinics today. As the onset of T2DM is asymptomatic for a large portion of the high-risk population, there is usually a latent phase before the confirmative diagnosis of T2DM during which risk factors for diabetic micro- and macro-vascular complications are markedly elevated (Harris and Eastman, 2000; Colagiuri and Davies, 2009). The diagnostic markers, such as fasting plasma glucose (FPG), oral glucose tolerance (OGT), and glycated hemoglobin (HbA1c), are the current gold standards for diagnosing T2DM in patients with hyperglycemia but their ability to detect T2DM is limited by the long asymptomatic phase of early T2DM (Lindström and Tuomilehto, 2003; Carson et al., 2010). As T2DM is a chronic condition that progresses over a long period and interventions with a proven beneficial effect are available. Early detection of the disease onset might allow immediate interventions to delay or prevent the disease progression (Colagiuri and Davies, 2009; Pan et al., 1997; Harris and Eastman, 2000; Knowler et al., 2002; investigators, 2006; Tuomilehto et al., 2001). We previously defined and characterized the critical transition state prior to the type 2 diabetes disease (Jin et al., 2017). Our analysis of a US state patients’ pre-disease clinical history identified a dynamic driver network (DDN) and an associated critical transition state 6 months prior to their first confirmative T2DM state. A meta-analysis reported that 3-months lifestyle interventions decreased the risk for diabetes from the end of intervention up to 10 years later (He et al., 2012). Therefore, the identification of pre-diabetic biomarkers in the circulation of asymptomatic patients could be highly beneficial. as a promising liquid biopsy utility for population health management.

Metabolomics is an emerging technology that allows the comprehensive characterization of metabolites in biological systems. It presents as a most recent addition to the omics family and provides complementary information to genomics and proteomics. Owing to the recent advances in analytical instrumentation and bioinformatic approach, metabolomic analysis based on liquid chromatography-high resolution mass spectrometry (LC/HRMS) has given rise to the in-depth profiling of diverse metabolites in a given biological system with superior sensitivity, thus becoming a powerful platform to discover novel biomarkers in close association with disease phenotypes for clinical applications (Xia et al., 2013). As metabolites are downstream end-products of genetic and proteomic regulations, the characterization of metabolome allows the effects of a plethora of pathological factors from vastly different origins to be determined in a single measurement, enabling the comprehensive understanding of molecular mechanisms underlying the disease phenotypes (Xia et al., 2013).

In this study, we aim to characterize and identify unique metabolic signatures using both targeted and global LC/HRMS for the early T2DM detection.

## 2 Materials and Methods

### 2.1 Study Population, Blood Collection, and Clinical Characteristic

Four independent cohorts were assembled for the study. The T2DM cohort included sera from 25 patients with T2DM and 25 healthy individuals for the biomarker discovery and sera from 15 patients with T2DM and 15 healthy controls for the testing. The Pre-T2DM cohort included sera from 76 patients with prediabetes and 62 healthy controls for the model training and sera from 35 patients with prediabetes and 27 healthy controls for the model testing. The study was approved by the Institutional Review Board of Teda Hospital and conducted in accordance with the Declaration of Helsinki. All experiments were performed in compliance with the requirements of the Human Ethics Procedures and Guidelines of the local government. Written informed consent was obtained from all participants. Patients with T2DM were diagnosed based on the American Diabetes Association criteria using FPG (FPG less than 100 mg/dL as non-diabetes, FPG between 100 and 125 mg/dL as prediabetes, and FPG greater than 125 mg/dL as diabetes). The serum samples were obtained by room temperature 30 min clotting of collected blood followed by 5 min centrifuge at 3,000 r/min. All serum samples were aliquoted and stored at −80°C prior to use. The clinical characteristics, including TC, TG, HDL-C, and LDL-C, were determined by Cobas C 311 blood analyzer from Roche (South San Francisco, CA, United States) by following the protocols from the manufacturer.

### 2.2 Global Metabolomics

#### 2.2.1 Metabolite Extraction

The metabolite extraction was conducted as previously described with slight modifications (Nemkov et al., 2017). Briefly, once thawed on ice, 10 µl of serum was extracted by 240 µl of prechilled methanol/acetonitrile/water (5:3:2, v/v) containing 3 stable isotope labeled amino acids as internal standards. Afterwards, the sample was vortexed rigorously for 30 min and centrifuged at 12,000 g for 10 min at 4°C. Thereafter, 200 µl of the supernatant was transferred into auto-sampler vial with micro-insert for LC/MS analysis.

#### 2.2.2 Global Metabolomic Profiling

The global metabolomic profiling was performed as previously described with slight modifications (Nemkov et al., 2017). Briefly, 10 µl of serum extract was injected onto a Kinetex C18 column (1.7 μm, 2.1 × 150 mm; Phenomenex, Torrance, CA) via a Vanquish UHPLC system (Thermo Fisher, San Jose, CA, United States). The mobile phase A was water with 0.1% formic acid, and mobile phase B was acetonitrile with 0.1% formic acid. The separation was carried out using isocratic elution with 5% B at a flow rate of 0.25 ml/min for a total run time of 3 min. The eluted metabolites were detected by a Q Exactive Plus mass spectrometer (Thermo Fisher) operated in full scan setup using both electrospray positive and negative modes, operating separately as two independent runs. The conditions of ionization source were set at 3.4 kV for spray voltage, 15 for sheath gas, 5 for aux gas, 325°C for capillary temperature, 55 for S-lens, and 250°C for vaporizer temperature. The MS spectra were acquired with 2 µscans using an AGC target of 1e^6^ and a resolution of 70,000 (FWHM at 200 m/z) from 60 to 900 m/z. The column oven was maintained at 25°C throughout the analysis. Representative chromatograms, with either positive or negative mode, were shown in Supplementary Figure S1.

#### 2.2.3 Data Pre-Processing

Following the analysis, the raw files were first converted into mzXML files by msconvert software from ProteoWizard Tools (http://proteowizard.sourceforge.net/tools.shtml). Subsequently, metabolic features with unique mass/charge ratio and retention time were extracted, aligned, quantified, grouped, and annotated using the XCMS online package (Smith et al., 2006). Afterwards, the obtained features underwent isotopic removal, blank subtraction, and missing value filtering and the QC-based signal drifting correction to normalize the undesired variations. The QC-based signal drifting correction based on locally estimated scatterplot smoothing was performed using StatTarget package (Luan et al., 2018).

#### 2.2.4 Metabolite Identification and Metabolic Network and Pathway Analysis

Identification of significant metabolite was conducted by matching the experimental retention time and accurate mass of the metabolic features against an in-house library containing 600 + authentic standards. The metabolic networking analysis and pathway enrichment analysis were implemented using the built-in function available on MetaboAnalyst 4.0 (Chong et al., 2019).

#### 2.2.5 QA/QC

The raw signals of internal standards were used to evaluate the extraction efficiency and instrumentation performance. The LC-MS system was conditioned with 10 consecutive injections of QC samples prior to the analysis of unknown samples. The batchwise systematic and random variations were evaluated by repeatedly injecting the QC samples that were spaced evenly. Signal drifting along the batch was corrected using a QC-based approach (Dunn et al., 2011). Between batches, the system was maintained and calibrated to meet the requirements for technical specifications.

#### 2.2.6 Metabolic Features

The data acquisitions were implemented in both electrospray ionization positive (ESI+) and negative (ESI−) modes to improve the coverage of serum metabolome. Following the data acquisition, the metabolic features were extracted, aligned, quantified, grouped, and annotated to generate a sample-feature intensity matrix with annotations for subsequent qualification. In total, 1958 and 2,892 metabolic features were detected in all serum samples from ESI+ and ESI−, respectively. After isotope removal ([M+1]/[M+2] ions), blank subtracted (signal-to-noise ≥5), and missing value filtered (≤ 20% missing values in QC samples), 751 and 1,003 metabolic features were qualified for ESI+ and ESI−, respectively. Upon normalized by QC-based signal drifting correction based on locally estimated scatterplot smoothing (LOESS), 323 and 295 metabolic features were reproducibly detected in QC replicates (coefficient variation ≤30%) for ESI+ and ESI-, respectively, and thus selected for the downstream data interpretation.

#### 2.2.7 Structure Determination

Metabolite biomarker identification was first performed as a Tier 1 or 2 identification with chemical standards according to MS1 (Schymanski et al., 2014). With tandem mass spectrometry (MS/MS, Thermo Q Exactive Plus) data of blood samples and manual review confirmation, the generated MS1/MS2 pairs were searched in the public databases: HMDB (http://www.hmdb.ca/), MoNA (http://mona.fiehnlab.ucdavis.edu/), MassBank (http://www.massbank.jp/), METLIN (https://metlin.scripps.edu), and NIST (https://www.nist.gov/). The metabolites of interest were procured and subjected to a Tier 1 identification comparing the retention time, MS1 and MS2 patterns with the biomarker candidates, using the same LCMS/MS protocol with the blood sample analysis.

### 2.3 Targeted Metabolomics

#### 2.3.1 Metabolite Extraction

For MS/MS analysis, 10 µl of serum, 10 µl of internal standard solution (AA: amino acids; AC: acylcarnitines; FA: fatty acids), 90 µl of extraction buffer, and 200 µl hexane were added for extraction. The sample was vortexed vigorously for 1 min and centrifuged at 12,000 g for 5 min 80 µl of lower layer was transferred into an auto-sampler vial for the analysis of amino acids and acylcarnitines. 180 µl of upper layer was transferred into another 1.5 ml centrifugal tube and was dried under nitrogen stream. The residue was reconstituted with 100 µl of derivatization buffer and incubated at 95°C for 15 min. After derivatization, 100 µl of neutralization buffer was added into each sample. The neutralized sample was vortexed vigorously for 1 min and centrifuged at 15,000 g for 3 min 100 µl of each reconstituted sample was transferred into an auto-sampler vial for the analysis of fatty acids.

#### 2.3.2 Targeted Metabolomic Profiling

##### 2.3.2.1 AA and AC

2 µl of serum extract was injected into a Vanquish UHPLC system (Thermo Fisher, San Jose, CA, United States). The mobile phase A was water with 0.5% formic acid and10 mM Ammonium Formate, mobile phase B was methanol with 0.5% formic acid and10 mM Ammonium Formate. The separation was carried out using isocratic elution with 50% B at a flow rate of 0.10 ml/min. The eluted metabolites were detected by an Altis mass spectrometer (Thermo Fisher) operated in SRM setup using electrospray positive mode. The conditions of ionization source were set at 3.5 kV for spray voltage, 20 for sheath gas, 5 for aux gas, 300°C for ion transfer tube temperature, 200°C for vaporizer temperature. The MS spectra were acquired using an cycle time of 0.8 and Q1 resolution (FWHM) of 0.7, Q3 resolution (FWHM) of 0.7, CID gas of 1.5, chromatographic peak width of 12. The column oven was maintained at 30°C throughout the analysis.

##### 2.3.2.2 FA

5 µL of serum extract was injected into a Vanquish UHPLC system (Thermo Fisher, San Jose, CA, United States). The mobile phase was methanol with 0.5% formic acid and 10 mM Ammonium Formate. The separation was carried out using isocratic elution with at a flow rate of 0.10 ml/min. The eluted metabolites were detected by an Altis mass spectrometer (Thermo Fisher) operated in SRM setup using APCI positive mode. The conditions of ionization source were set at 6 µA for ion discharge current, 20 for sheath gas, 5 for aux gas, 300°C for ion transfer tube temperature, 300°C for vaporizer temperature. The MS spectra were acquired using an cycle time of 1 and Q1 resolution (FWHM) of 0.7, Q3 resolution (FWHM) of 0.7, CID gas of 1.5, chromatographic peak width of 12. The column oven was maintained at 30°C throughout the analysis.

Multiple reaction monitoring (MRM) is the most common targeted method for quantitation of analytes by LC/MS/MS. In MRM, ions are selected to make it through the first quadrupole and into the collision cell. In this study, the transition from precursor/parent ion to product/daughter ions is referred to as an ion transition (Supplementary Table S1).

#### 2.3.3 Statistical Analysis

The normalized intensity of metabolic features from various samples were log-transformed and auto scaled prior to the statistical analysis. Multivariate and univariate statistical analysis were performed using MetaboAnalyst 4.0 (https://www.metaboanalyst.ca/) (Chong et al., 2019). A global false discovery rate of 5% was applied to correct for the errors from multiple hypothesis testing. All statistical analyses were preformed using R packages (Tibshirani, 2006; Team, 2008). We have applied power analysis (Tibshirani, 2006) to determine the sample size for the testing of the T2DM and Pre-T2DM models (Supplementary Figures 2A,B).

#### 2.3.4 Machine Learning Approach Evaluation

The learning procedure was empirically compared against a number of standard multivariate algorithms, including Random Forest (Breiman, 2001), Elastic Net (Zou and Hastie, 2005) and XGboost (Chen and Guestrin, 2016). All algorithms were evaluated using the same two-layer leave-one-subject-out cross validation strategy.

## 3 Results

### 3.1 Study Design

T2DM is a highly prevalent chronic metabolic disorder characterized by hyperglycemia. We implemented both the global and targeted mass spectrometry (MS)-based metabolomics to advance T2DM research. The time associated with traditional chromatographic methods for resolving metabolites prior to mass analysis has limited the potential to perform large-scale, highly powered metabolomics studies, and clinical applications. Therefore, we applied a 3-min method (Nemkov et al., 2017) for the rapid profiling of central metabolic pathways through UHPLC/MS. Moreover, we applied targeted metabolomics to profile amino acids, acylcarnitines, and fatty acids as several studies suggest a central role for oxidative stress in the pathogenesis of the disease. The short-, medium-, and long-chain acylcarnitines are a family of metabolites known to be dysregulated in T2DM (Bene et al., 2018), linked to peripheral insulin resistance. These acylcarnitines have an indispensable role in lipid metabolism via their involvement in the less severe disruptions in *β*-oxidation of long-chain fatty acids in T2DM. The dysregulated fatty acid metabolism along with tissue lipid accumulation is generally assumed to be associated in the development of insulin resistance and T2DM (Sears and Perry, 2015). Increased levels of total plasma free fatty acid and branched-chain amino acids (BCAAs) are associated with T2DM pathogenesis (Sjögren et al., 2021).

**FIGURE 1:**
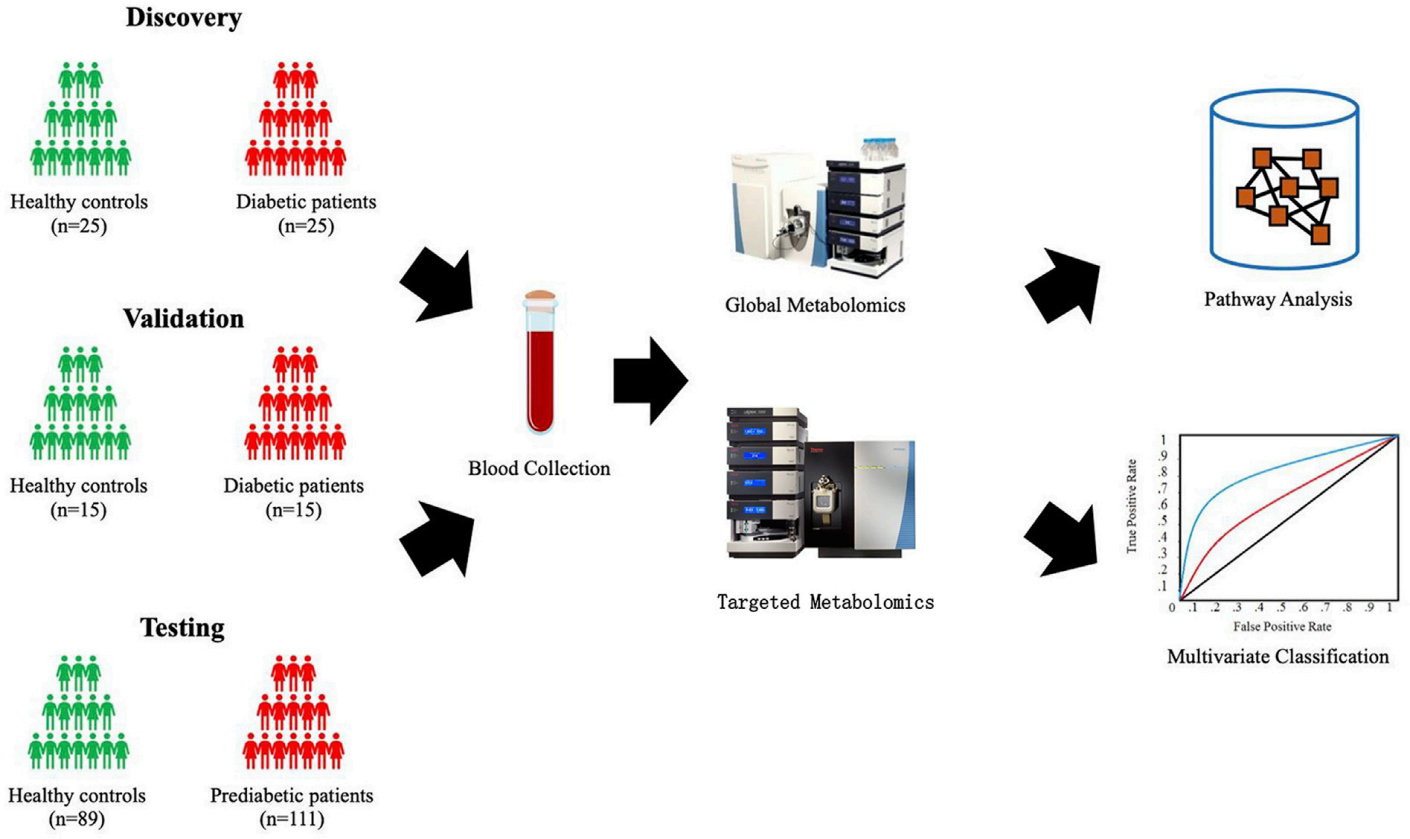
Schematic study design.

Shown in Figure 1, four independent cohorts were assembled for the study. The T2DM cohort included sera from 25 patients with T2DM and 25 healthy individuals for the biomarker discovery and sera from 15 patients with T2DM and 15 healthy controls for the testing. The Pre-T2DM cohort included sera from 76 patients with prediabetes and 62 healthy controls for the model training and sera from 35 patients with prediabetes and 27 healthy controls for the model testing.

### 3.2 Sample Demographics and Clinical Characteristics

The sample demographics and clinical characteristics are summarized in Table 1. T2DM associated clinical characteristics were analyzed: fasting plasma glucose (FPG), high-density lipoprotein cholesterol, triglyceride, and creatinine were found to be statistically significant (*p* < 0.05) between case and control groups, whereas the other clinical characteristics, including low density lipoprotein (LDL) cholesterol, total cholesterol, and serum creatinine, were insignificant (*p* > 0.05) between case and control groups among all studied cohorts.

**TABLE 1:** Demographic table.

Characteristic	Diabetic Cohort						Pre-diabetic Cohort					
	Training			Testing			Training			Testing		
	Non-Diabetes (n = 25)	Diabetes (n = 25)	*p* value	Non-Diabetes (n = 15)	Diabetes (n = 15)	*p* value	Non-Diabetes (n = 62)	Pre-Diabetes (n = 76)	*p* value	Non-Diabetes (n = 27)	Pre-Diabetes (n = 35)	*p* value
Age (year)	45.9 (1.1)	52.2 (4.7)	0.045	69.5 (3.2)	67.2 (5.9)	0.192	54.5 (5.2)	53.8 (6.2)	0.441	54.3 (4.2)	55.5 (6.8)	0.404
Gender												
Male	10 (40.0%)	15 (60.0%)		5 (33.3%)	10 (66.7%)		29 (46.8)	53 (69.7)		7 (25.9)	21 (60)	
Female	15 (60.0%)	10 (40.0%)		10 (66.7%)	5 (33.3%)		33 (53.2)	23 (30.3)		20 (74.1)	14 (40)	
FPG (mM)	5.1 (1.8)	8.9 (3.0)^***^	7.1 × 10^−10^	5.1 (0.3)	9.6 (2.2)^***^	1.7 × 10^−6^	5.2 (0.3)	6 (0.3)^***^	3.7 × 10^−5^	5.2 (0.6)	6.1 (0.4)^***^	2.9 × 10^−6^
TC (mM)	4.9 (0.8)	5.1 (1.2)	0.72	9.6 (2.2)	4.7 (1.1)	0.426	4.7 (0.9)	4.8 (1)	0.319	5.2 (0.9)	4.9 (1)	0.231
Creatinine (mM)	65.9 (13.7)	66.8 (19.9)	0.98	68.2 (11.0)	68 (14.1)	0.485	68 (14.1)	71.2 (13.5)	0.175	64.1 (10.8)	69.9 (13.1)	0.069
TG (mM)	1.3 (0.6)	2.3 (1.0)^***^	4.5 × 10^−4^	1.4 (0.5)	1.4 (0.6)	8.4 × 10^−5^	1.4 (0.6)	1.8 (1.1)^**^	0.03	1.3 (0.5)	1.8 (0.9)^**^	0.009
HDL-C (mM)	1.4 (0.6)	1.1 (0.4)	0.049	1.4 (0.3)	1.3 (0.4)	0.0236	1.3 (0.3)	1.2 (0.4)^***^	0.249	1.5 (0.4)	1.1 (0.3)^***^	2.8 × 10^−4^
LDL-C (mM)	3.2 (0.8)	3.1 (1.1)	0.791	3.0 (0.7)	3.0 (1.0)	0.86	3 (0.8)	3 (1)	0.93	3.4 (0.9)	3.2 (1)	0.299

All values are presented as mean (SD) except for gender where percentage is applied. The *p* values were determined by Mann-Whitney U test and classified into several categories based on following criteria by comparing against the control group: *: *p* < 0.05; **: *p* < 0.01; ***: *p* < 0.001. FPG, fast plasma glucose; TC, total cholesterol; HDL-C, high density lipoprotein cholesterol; LDL-C, low density lipoprotein cholesterol; TG, triglycerides.

### 3.3 Identification of Metabolic Signature for T2DM

**FIGURE 2:**
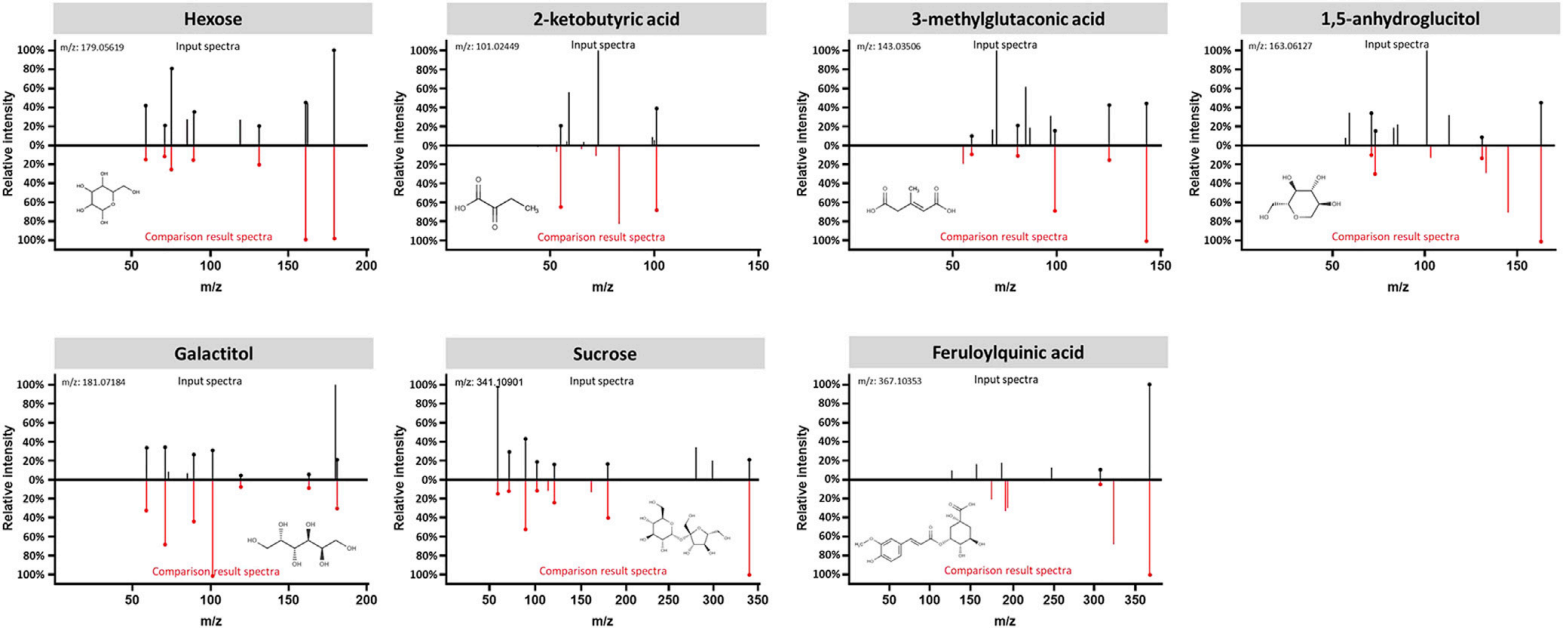
Structural identification of the biomarker compounds discovered from global metabolomics analysis: Hexose, 2-ketobutyric acid, 3-methylglutaconic acid, 1,5-anhydroglucitol, Galactitol, Sucrose, and Feruloylquinic acid. Measured MS/MS spectral fragmentation profiles (top, in black) matching procured chemical standards (bottom, in red) profiled with the same LCMS protocol.

**FIGURE 3:**
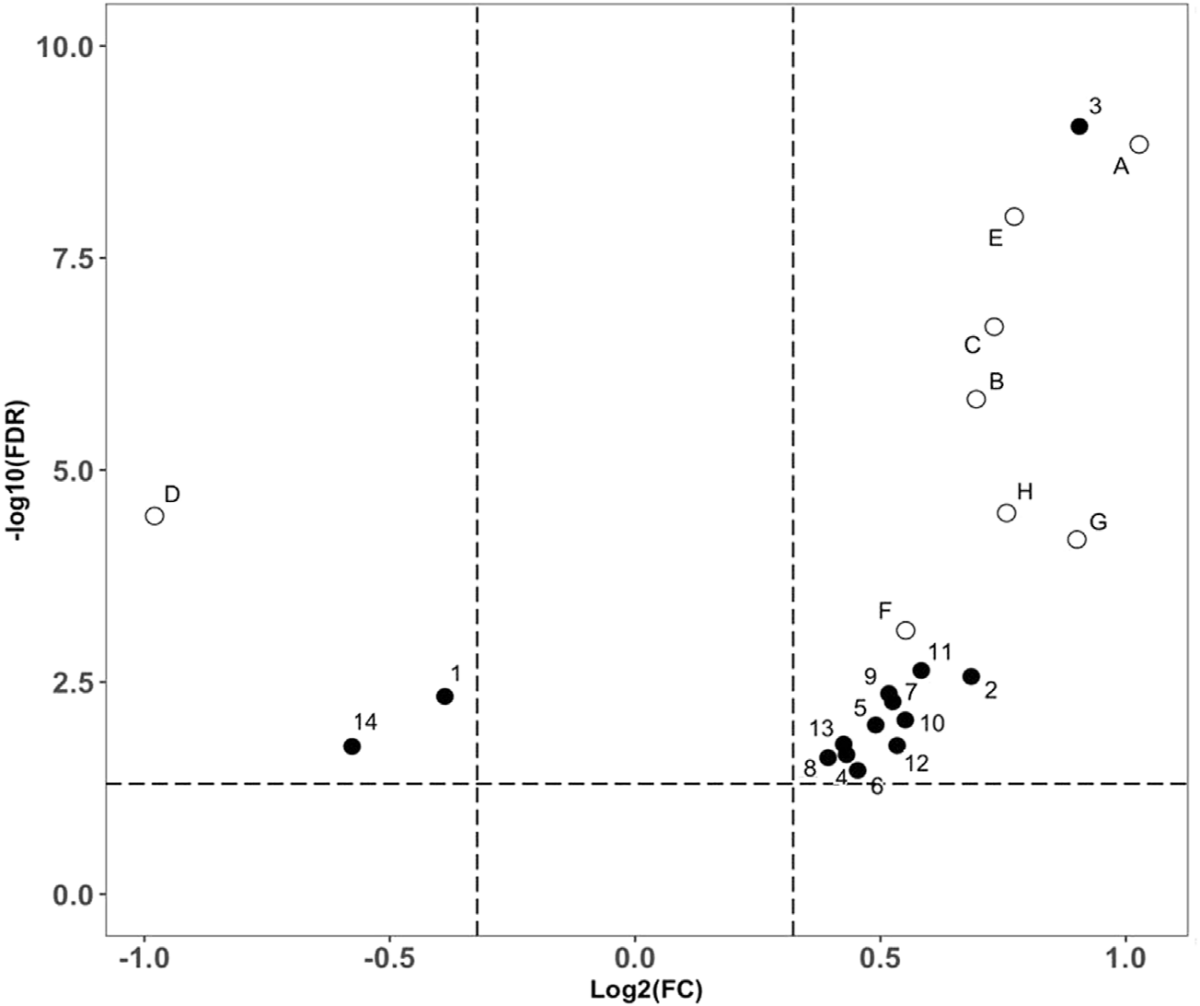
Volcano plot analysis of the biomarker compounds revealed in our discovery analysis with the T2DM training cohort. Filled circles representing compounds discovered from the targeted metabolomics analysis. **(A)**: Glucose ([M + Na]+), **(B)**: 2-Ketobutyric acid, **(C)**: 3-Methylglutaconic acid, **(D)**: 1,5-anhydroglucitol, **(E)**: Glucose ([M-H]-), **(F)**: Galactitol, **(G)**: Sucrose, **(H)**: Feruloylquinic acid. Open circles representing compounds discovered from the global metabolomics analysis, 1: Pyroglutamic acid, 2: Ornithine, 3: Hexose, 4: Valine, 5: Leucine/Isoleucine, 6: Tyrosine, 7: Phenylalanine, 8: Tryptophan, 9: C16-Carnitine, 10: C14-Carnitine, 11: C5DC-Carnitine/C6OH-Carnitine, 12: C5OH-Carnitine, 13: Tritriacontanoic Acid (33:0) Butyl Ester, and 14: Dotriacontanoic Acid (32:0) Butyl Ester.

The data interpretation was performed in a stepwise manner using a suite of bioinformatic approaches. The Mann-Whitney U test with a 5% false discovery rate was used to determine the statistical significance of individual metabolic features by comparing the case to control groups from the T2DM training cohort. From the global metabolomic analysis, 12 validated features were putatively identified, with MS1 Tier 1 identification, by matching the retention times and accurate masses against an in-house library containing 600+ authentic standards. 8/12 were structurally determined (Figure 2, with Tier 2 identification) were determined: hexose [M + Na]^+^, hexose [M-H]^−^, 1,5-anhydroglucitol, feruloylquinic acid, galactitol, 2-ketobutyric acid, 3-methylglutaconic acid, and sucrose. From the targeted metabolomic analysis, 14 were identified: pyroglutamic acid, ornithine, hexose, valine, leucine/isoleucine, tyrosine, phenylalanine, tryptophan, C16-carnitine, C14-carnitine, C5DC-carnitine/C6OH-carnitine, C5OH-carnitine, tritriacontanoic acid (33:0) butyl ester, and dotriacontanoic acid (32:0) butyl ester. These 22 metabolites constitute the T2DM biomarker panel for the following analyses. The univariate statistics of the T2DM panel biomarkers are illustrated in Figure 3.

**FIGURE 4:**
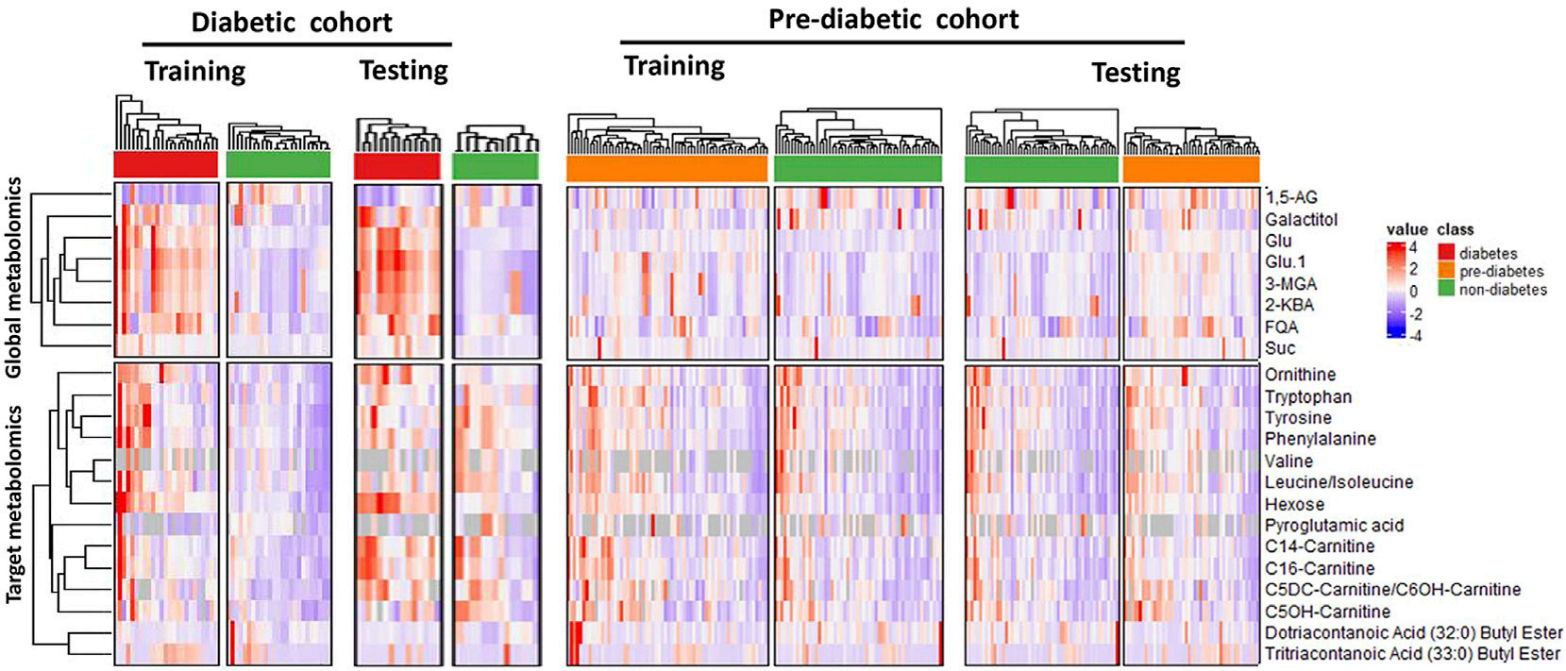
Unsupervised clustering (heatmap analysis) of 22 classifying metabolites (8 from global metabolomics and 14 from targeted metabolomics) reveals distinct metablic patterns separating diabetic, pre-diabetic samples from healthy controls. Abbreviations are as follows: 1,5-AG, 1,5-anhydroglucitol; FQA, Feruloylquinic acid; Glu, Glucose; 3-MGA, 3-methylglutaconic acid; 2-KBA, 2-ketobutyric acid and Suc, Sucrose.

With unsupervised clustering, the heatmap revealed a distinct T2DM pattern of these 22 compound biomarkers (Figure 4), separating T2DM subjects from the non-diabetic controls in both training and testing cohorts.

### 3.4 Exploration of the T2DM Metabolic Panel to Assess Prediabetic Patients in the General Population

**FIGURE 5:**
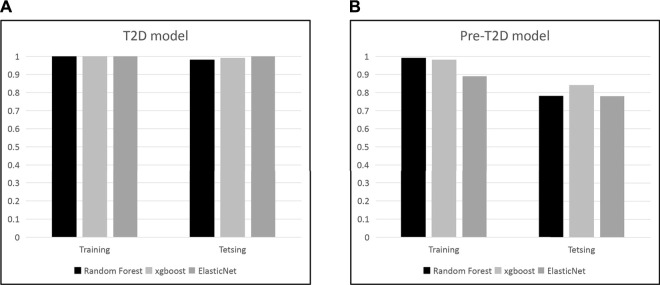
Development of T2DM and Pre-T2DM models with different machine learning approaches.

We set to apply the T2DM panel to assess those who are at high risk of developing T2DM. Not as a pronounced pattern of the T2DM panel seen in the T2DM cohort, the pattern of these 22 compound biomarkers (Figure 4) persists in the Pre-T2DM cohort, separating prediabetic subjects from the non-diabetic controls in both training and testing cohorts. We applied different machine learning algorithms, including Random Forest, XGBoost and Elastic Net, to these datasets for the T2DM assessment. For the T2DM dataset, we developed the model with the T2DM training set which achieved similar superb performance for the testing set: Random Forest, ROC AUC 0.98; XGBoost, ROC AUC 0.99; Elastic Net, ROC AUC 1.000 (Figure 5A). For the Pre-T2DM cohort, we developed the model with the Pre-T2DM training set and found XGBoost performed the best for the Pre-T2DM testing set: Random Forest, ROC AUC 0.78; XGBoost, ROC AUC 0.84; Elastic Net, ROC AUC 0.78 (Figure 5B). Therefore, XGBoost Pre-T2DM model would be the model for the analysis of the general population at risk for the T2DM at early prediabetic stage.

**FIGURE 6:**
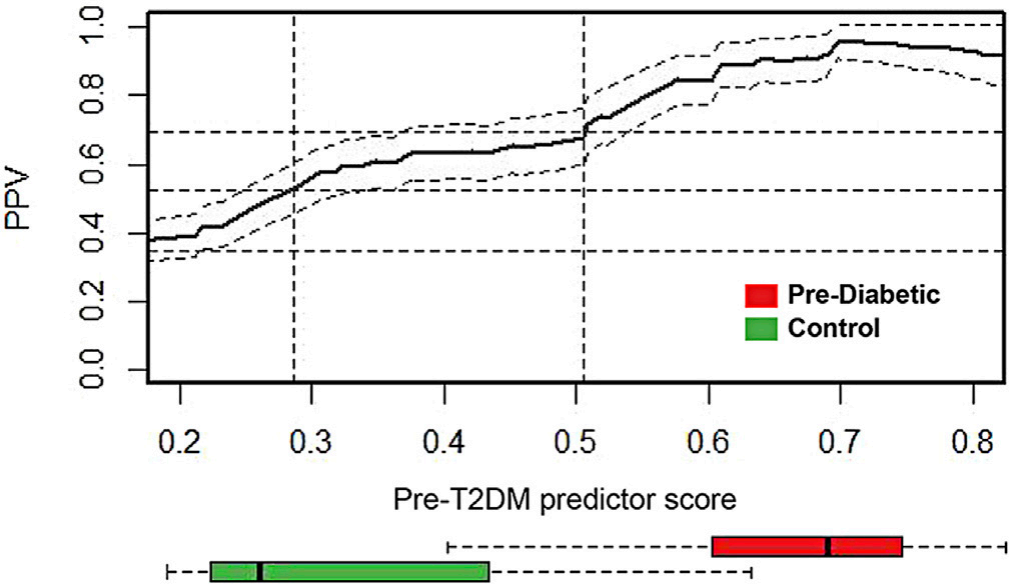
Prevalence-corrected positive predictive values (PPV) was plotted as a function of Pre-T2DM predictor score for the Pre-T2DM cohort samples. Horizontal dashed lines identify the average population risk of 34.8%, and relative risks of 1.5X (52.2%) and 2X (69.6%). Vertical dashed lines identify corresponding predictor scores. The confidence interval about the PPV curve (gray shaded area) was estimated using all Pre-T2DM subjects. Confidence intervals about the PPV were calculated with the normal approximation of the error for binomial proportions. Box plots at the foot of the figure correspond to the distributions of predictor scores for prediabetic and control subjects. The PPV curve and the box plots share the same predictor score axis.

Cross-sectional retrospective cohort analysis (Le et al., 2019) of the 2003–2014 National Health and Nutrition Examination Survey (NHANES) reported the prevalence of prediabetes (34.8%). Shown in Figure 6, the prevalence adjusted positive predictive values (PPV), a measure of clinical risk, is shown as a function of XGBoost Pre-T2DM model predictor score. Stratification of subjects with increasing predictor scores occurs as positive predictive values (PPV) from a background value (population rate of 34.8%) to relative risks of 1.5X (52.2%) and 2X (69.6%) (dashed lines) and higher. The distribution of Pre-T2DM predictor score values for subjects color-coded (green, controls; red, Pre-T2DM) are shown in box plots in Figure 6. Using the risk curve in Figure 6, Pre-T2DM subjects were identified as high or low risk according to a predictor score cutoff corresponding to 2X relative risk (PPV of 69.6%, predictor score = 0.505).

## 4 Discussion

Early detection of T2DM remains a challenge in current clinical practice. Diagnostic markers including FPG, OGT, and Hb1Ac are gold standards in identifying impaired glucose disposition and poor glycose management in patients with T2DM. However, a long latent phase of T2DM onset occurs asymptomatically before hyperglycemic symptoms are manifested (Lindström and Tuomilehto, 2003; Carson et al., 2010). The current screening test utilizes several risk factors such as metabolic syndrome and family history for early assessment of T2DM, which are short in both sensitivity and specificity. Moreover, T2DM is a chronic condition that progresses over a long time, and early detection of T2DM might allow immediate interventions such as dietary control, weight loss, and glucose-lowering medications for effective management of the disease (Pan et al., 1997; Knowler et al., 2002; investigators, 2006; Tuomilehto et al., 2001). Aiming for the early detection of general population at high risk of T2DM, we employed an omics-based approach integrating global metabolomic profiling and bioinformatic analysis to identify potential pre-diabetic biomarkers in blood. The efforts from the T2DM metabolic panel discovery, testing, and application to prediabetic cohorts to case find subjects at high risk for T2DM led to the identification of 22 metabolites in circulation with a unique metabolic pattern associated with T2DM risk. The multivariate analysis based on those metabolites indicated the presence of significantly dysregulated metabolism in patients with early progressing T2DM, which was differentiative between pre-diabetic and non-diabetic subjects. Longitudinal measurement of the metabolic panel thus might offer an opportunity for assessing diabetic onset before the development of relevant clinical signs and symptoms.

Our study also identified 22 metabolites that were significantly altered in T2DM, and models were developed in risk stratification to case find the general population at risk of the onset of prediabetes. Hexose represents monosaccharides with a six-carbon scaffold, and glucose is known to constitute over 95% of hexose content in humans. Sucrose is a nonreducing disaccharide composed of glucose and fructose linked via their anomeric carbons. Increased dietary intake of sucrose is considered a risk factor for diabetic pathogenesis, and a recent study has discovered that the dietary replacement of sucrose by fructose can significantly improve glucose disposition and insulin sensitivity (Evans et al., 2017). Our study revealed significantly increased circulating hexose and sucrose induced by defective insulin secretion and impaired insulin sensitivity in T2DM, consistent with disease pathophysiology (Gonzalez-Franquesa et al., 2016; Klein and Shearer, 2016; Arneth et al., 2019; Sun et al., 2019). 2–KBA is produced by amino acid catabolism (threonine and methionine) and glutathione anabolism (cysteine formation pathway) and is metabolized to propionyl-CoA and carbon dioxide (Gall et al., 2010; Syed Ikmal et al., 2013). During the formation of 2–KBA, 2-hydroxybutyrate (2–HBA) is formed as a by-product, and mounting evidence has demonstrated that circulating 2-HBA is inversely correlated with insulin sensitivity and elevated 2-HBA is an early marker for both insulin resistance and impaired glucose regulation independent of sex, age, and BMI (Gall et al., 2010; Syed Ikmal et al., 2013). 2–KBA can also be produced in the conversion of cystathionine to cysteine. In the presence of enhanced oxidative stress in T2DM, a metabolic shift occurs by stimulating homocysteine production from transmethylation of methionine to transsulfuration of homocysteine to produce cystathionine and cysteine for meeting the increased demand of glutathione, the primary cytoplasmic antioxidant (Ratnam et al., 2002; Schalinske, 2003). Cystathionine is a metabolic intermediate formed by the condensation of serine and homocysteine via the catalysis of cystathionine *β*-synthase (CBS) for homocysteine transsulfuration. Markedly elevated transcriptional expression and enzymatic activity of CBS were observed in a streptozotocin-induced diabetic rat model, and the administration of regulatory hormones such as insulin was able to revert the elevation of CBS to result in the attenuation of diabetic phenotype (Ratnam et al., 2002). Our study discovered the upregulation of 2-KBA and cystathionine in T2DM, further confirming their mechanistic roles in glycemic regulation. 1,5-AG is a metabolically inert polyol that competes with glucose for reabsorption in the kidneys and has been validated as a marker of short-term glycemic control. Its level is inversely correlated with glucose level in blood circulation, and reduced plasma 1,5-AG is a sensitive marker for increased urinary excretion of glucose (Dungan, 2008; Kim and Park, 2013). Several studies have proposed 1,5-AG as a short-term retrospective marker for monitoring glucose excursions (Dungan, 2008; Kim and Park, 2013). Our results revealed the downregulation of 1,5-AG in T2DM, again validating its utility as a biomarker for monitoring short-term glycemic management. FQA is a subclass of chlorogenic acids (CGAs) that are esters formed between caffeic and quinic acids. CGA represents an abundant group of plant polyphenols present in the human diet. *In-vitro* and *in-vivo* studies have indicated their potent anti-oxidative and anti-inflammation activities by prolonging the lifetime of the phenoxyl radical and upregulating the pro-inflammatory cytokines at transcriptional level (Liang and Kitts, 2016). Results from epidemiological studies have suggested that the consumption of beverages containing CGA is associated with reduced risks of developing chronic diseases such as T2DM (Tajik et al., 2017). Our results illustrated a significant upregulation of FQA in T2DM, suggesting a surging demand for antioxidants to counteract increased oxidative stress in diabetes. Our targeted analysis revealed branched chain amino acid (BCAA) valine and leucine/isoleucine, and aromatic amino acid (AAA) phenylalanine, tyrosine and tryptophan as biomarkers for T2DM. Elevated plasma concentrations of BCAA and AAA circulate up to 10 years prior to a diagnosis of T2DM, hence interest in their role as biomarkers for insulin resistance and T2DM (Cheng et al., 2012; Saleem et al., 2019). Our results of C16/C14/C5DC/C6OH/C5OH carnitine alterations in serum concentrations in both T2DM and prediabetic states are in line with previous findings about the role of mitochondrial function in the complex pathogenesis of type 2 diabetes. The mitochondrial function seems to play a significant role in the complex pathogenesis of insulin resistance (Kelley et al., 2002; Koves et al., 2008; Mihalik et al., 2010). Recent studies suggest that concentrations of various acylcarnitines are associated with insulin resistance and T2D (Adams et al., 2009; Huffman et al., 2009; Tai et al., 2010; Ha et al., 2012) and incomplete fatty acid oxidation results in elevated acylcarnitine concentrations (Koves et al., 2005). In addition, our study also identified several novel markers, including 3-MGA, galactitol, ornithine, pyroglutamic acid, and dotria/tritriacontanoic acid (32:0/33:0) butyl esters with unknown implications in T2DM and further investigations on their pathological implications might uncover novel molecular mechanisms to provide more comprehensive understanding of T2DM pathophysiology and guide the development of new therapeutic with better efficacy for treating T2DM.

Despite the strengths of our study results, these findings need to be evaluated in the context of the study limitations. A prospective longitudinal high resolution sampling cohort assembly with the emerging T2DM as a clinical outcome can validate the clinical utility of our T2DM metabolic panel to allow the early detection of the disease onset for proactive intervention.

In conclusion, we presented a metabolomics-based discovery study with T2DM and prediabetic subjects. Using sera from four independent cohorts, a panel of metabolic biomarker candidates was discovered from T2DM cohort and tested with Pre-T2DM cohort to risk stratify the general population to case find the high risk subjects of emerging T2DM. Application of this predictor should enable early and sensitive detection of generation population at risk of T2DM. This early detection may improve clinical outcomes through increased clinical surveillance as well as accelerate the development of clinical interventions for T2DM prevention.

## Data Availability

The raw data supporting the conclusion of this article will be made available by the authors, without undue reservation.
