# Supplementary material for: Serological Phenotyping Analysis Uncovers a Unique Metabolomic Pattern Associated With Early Onset of Type 2 Diabetes Mellitus
Source: Front Mol Biosci. 2022 Apr 8;9:841209. doi: 10.3389/fmolb.2022.841209 (PMC9024215; doi:10.3389/fmolb.2022.841209)
Supplement: Supplementary file 1 [file DataSheet1.PDF]

Supplementary Material

Supplemental Table 1. MRM transition table for targeted analysis

Compound	Retention Time (min)	RT Window (min)	Polarity	Precursor (m/z)	Product (m/z)	Collision Energy (V)	RF Lens (V)
Glycine	0.45	0.9	Positive	76.374	30.2	10.253	30
13C-15N-Glycine	0.45	0.9	Positive	78.374	32.2	10.253	30
Alanine	0.45	0.9	Positive	90.374	44.146	10.253	30
D4-Alanine	0.45	0.9	Positive	94.374	48.146	10.253	30
Pyroglutamic acid	0.45	0.9	Positive	130	84	16.8	30
Proline	0.45	0.9	Positive	116.152	70.022	15.41	65.101
13C5-15N-Proline	0.45	0.9	Positive	122.152	75.02	15.41	65
Valine	0.45	0.9	Positive	118.382	72.025	10.253	30
D8-Valine	0.45	0.9	Positive	126.382	80.025	10.253	30
Leucine/Isoleucine	0.45	0.9	Positive	132.037	86.076	10.253	34.697
D3-Leucine	0.45	0.9	Positive	135.037	89.076	10.253	35
Ornithine	0.45	0.9	Positive	133.333	70.025	16.573	38.404
D2-Ornithine	0.45	0.9	Positive	135.333	72.025	16.573	39
Glutamine/Lysine	0.45	0.9	Positive	147.1	84	25	38.652
Asparagine	0.45	0.9	Positive	133.06	87.07	20	38.652
Citrulline	0.45	0.9	Positive	176.383	113.005	15.461	41.618
D2-Citrulline	0.45	0.9	Positive	178.383	115.005	15.461	42
Methionine	0.45	0.9	Positive	150.332	132.955	10.253	34.697
D3-Methionine	0.45	0.9	Positive	153.332	135.955	10.253	38
Histidine	0.45	0.9	Positive	156.1	110	12	50
Arginine	0.45	0.9	Positive	174.926	70.025	22.034	53.483
D4-Arginine	0.45	0.9	Positive	180.23	75.15	22.034	53
Tryptophan	0.45	0.9	Positive	205.1	188.3	16	55
Phenylalanine	0.45	0.9	Positive	166.017	120.025	12.326	42.36
13C6-Phenlalanine	0.45	0.9	Positive	172.017	126.025	12.326	42
Threonine	0.45	0.9	Positive	120.1	74	15	38
Serine	0.45	0.9	Positive	106	60	18	30
Tyrosine	0.45	0.9	Positive	182.028	135.984	12.781	43.843
13C6-Tyrosine	0.45	0.9	Positive	188.028	141.984	12.781	44
Hexose	0.45	0.9	Positive	203.009	23.325	10.253	59
D7-Glucose	0.45	0.9	Positive	210.009	23.325	10.253	59
Creatinine	0.45	0.9	Positive	114.352	86.04	12.073	56

D3-Creatinine	0.45	0.9	Positive	117.383	89.111	11.315	60
Uric Acid	0.45	0.9	Positive	169.039	141	16.978	76
15N2-Uric Acid	0.45	0.9	Positive	171.039	143	16.978	76
C0-Carnitine	0.45	0.9	Positive	162.382	85	19.303	60
D9-C0-Carnitine	0.45	0.9	Positive	171.382	85	19.303	60
C2-Carnitine	0.45	0.9	Positive	203.895	85	19.253	58.18
D3-C2-Carnitine	0.45	0.9	Positive	207.108	85	19.253	58
C3-Carnitine	0.45	0.9	Positive	218.108	85	19.101	100
D3-C3-Carnitine	0.45	0.9	Positive	221.108	85	19.101	100
C4-Carnitine	0.45	0.9	Positive	232.119	85	17.837	63
D3-C4-Carnitine	0.45	0.9	Positive	235.119	85	17.837	63
C5-Carnitine	0.45	0.9	Positive	246.27	85	20.315	66
C6-Carnitine	0.45	0.9	Positive	260.3	85	20.315	66
D9-C5-Carnitine	0.45	0.9	Positive	255.27	85	20.315	66
C8:1-Carnitine	0.45	0.9	Positive	286.208	85	21.781	74.742
C8-Carnitine	0.45	0.9	Positive	288.208	85	21.781	74.742
D3-C8-Carnitine	0.45	0.9	Positive	291.208	85	21.781	75
C10-Carnitine	0.45	0.9	Positive	316.33	85	21.781	75
C10:1-Carnitine	0.45	0.9	Positive	314.33	85	21.781	75
C10:2-Carnitine	0.45	0.9	Positive	312.33	85	21.781	75
C12-Carnitine	0.45	0.9	Positive	344.267	85	23.652	85.371
C12:1-Carnitine	0.45	0.9	Positive	342.267	85	23.652	85.371
D9-C12-Carnitine	0.45	0.9	Positive	353.267	85	23.652	85
C12:1OH-Carnitine	0.45	0.9	Positive	358.267	85	27.747	99.708
C14:1OH-Carnitine	0.45	0.9	Positive	386.359	85	27.747	99.708
D3-C16OH-Carnitine	0.45	0.9	Positive	419.389	85	27.747	100
C14-Carnitine	0.45	0.9	Positive	372.359	85	25.27	119.236
C14:1-Carnitine	0.45	0.9	Positive	370.45	85	25.27	119
C14:2-Carnitine	0.45	0.9	Positive	368.359	85	25.27	119
D9-C14-Carnitine	0.45	0.9	Positive	381.359	85	25.27	119
C16-Carnitine	0.45	0.9	Positive	400.389	85	26.129	94
C16:1-Carnitine	0.45	0.9	Positive	398.4	85	26.129	94
D3-C16-Carnitine	0.45	0.9	Positive	403.389	85	26.129	94
C18-Carnitine	0.45	0.9	Positive	428.389	85	27.09	102
C18:1-Carnitine	0.45	0.9	Positive	426.4	85	27.09	102
C18:2-Carnitine	0.45	0.9	Positive	424.4	85	27.09	102
D3-C18-Carnitine	0.45	0.9	Positive	431.389	85	27.09	102
C5DC-Carnitine/C6OH-Carnitine	0.45	0.9	Positive	276.2	85	22.843	70

D3-C5DC-Carnitine	0.45	0.9	Positive	279.2	85	22.843	70
C5OH-Carnitine	0.45	0.9	Positive	262.2	85	22.084	59
D3-C5OH-Carnitine	0.45	0.9	Positive	265.2	85	22.084	59
Lauric acid (12:0) Butyl Ester	0.45	0.9	Positive	269.2	213.2	11	90
Myristic Acid (14:0) Butyl Ester	0.45	0.9	Positive	285.3	229.3	11	90
Palmitoleic Acid (16:1) Butyl Ester	0.45	0.9	Positive	311.3	255.3	11	90
Palmitic Acid (16:0) Butyl Ester	0.45	0.9	Positive	313.3	257.3	11	90
Linolenic Acid (18:3) Butyl Ester	0.45	0.9	Positive	335.4	279.4	11	90
Linoleic Acid (18:2) Butyl Ester	0.45	0.9	Positive	337.4	281.4	11	90
Oleic Acid (18:1) Butyl Ester	0.45	0.9	Positive	339.4	283.4	11	90
Stearic Acid (18:0) Butyl Ester	0.45	0.9	Positive	341.4	285.4	11	90
Eicosapentaenoic Acid (20:5) Butyl Ester	0.45	0.9	Positive	359.4	303.4	11	90
Arachidonic Acid (20:4) Butyl Ester	0.45	0.9	Positive	361.4	305.4	11	90
Eicosatrienoic Acid (20:3) Butyl Ester	0.45	0.9	Positive	363.4	307.4	11	90
Eicosatrienoic Acid (20:2) Butyl Ester	0.45	0.9	Positive	365.4	309.4	11	90
Eicosatrienoic Acid (20:1) Butyl Ester	0.45	0.9	Positive	367.4	311.4	11	90
Arachidic Acid (20:0) Butyl Ester	0.45	0.9	Positive	369.4	313.4	11	90
Docosahexaenoic Acid (22:6) Butyl Ester	0.45	0.9	Positive	385.5	329.5	11	90
Docosapentaenoic Acid (22:5) Butyl Ester	0.45	0.9	Positive	387.5	331.5	11	90
Docosatetraenoic Acid (22:4) Butyl Ester	0.45	0.9	Positive	389.5	333.5	11	90
Docosatrienoic Acid (22:3) Butyl Ester	0.45	0.9	Positive	391.5	335.5	11	90

Docosadienoic Acid (22:2) Butyl Ester	0.45	0.9	Positive	393.5	337.5	11	90
Erucic Acid (22:1) Butyl Ester	0.45	0.9	Positive	395.5	339.5	11	90
Docosanoic Acid (22:0) Butyl Ester	0.45	0.9	Positive	397.5	341.5	11	90
D39-Arachidic Acid (20:0) Butyl Ester	0.45	0.9	Positive	408.7	352.7	11	90
Nervonic Acid (24:1) Butyl Ester	0.45	0.9	Positive	423.6	367.6	11	90
Lignoceric Acid (24:0) Butyl Ester	0.45	0.9	Positive	425.6	369.6	11	90
Pentacosanoic Acid (25:0) Butyl Ester	0.45	0.9	Positive	439.6	383.6	11	90
Hexacosanoic Acid (26:0) Butyl Ester	0.45	0.9	Positive	453.6	397.6	11	90
Heptacosanoic Acid (27:0) Butyl Ester	0.45	0.9	Positive	467.6	411.6	11	90
Octacosanoic Acid (28:0) Butyl Ester	0.45	0.9	Positive	481.6	425.6	11	90
Nonacosanoic Acid (29:0) Butyl Ester	0.45	0.9	Positive	495.6	439.6	11	90
Triacontanoic Acid (30:0) Butyl Ester	0.45	0.9	Positive	509.7	453.7	11	90
Dotriacontanoic Acid (32:0) Butyl Ester	0.45	0.9	Positive	537.7	481.7	11	90
Tritriacontanoic Acid (33:0) Butyl Ester	0.45	0.9	Positive	551.7	495.7	11	90
Tetratriacontanoic Acid (34:0) Butyl Ester	0.45	0.9	Positive	565.7	509.7	11	90
Hexatriacontanoic Acid (36:0) Butyl Ester	0.45	0.9	Positive	593.7	537.7	11	90
Heptatriacontanoic Acid (37:0) Butyl Ester	0.45	0.9	Positive	607.7	551.7	11	90

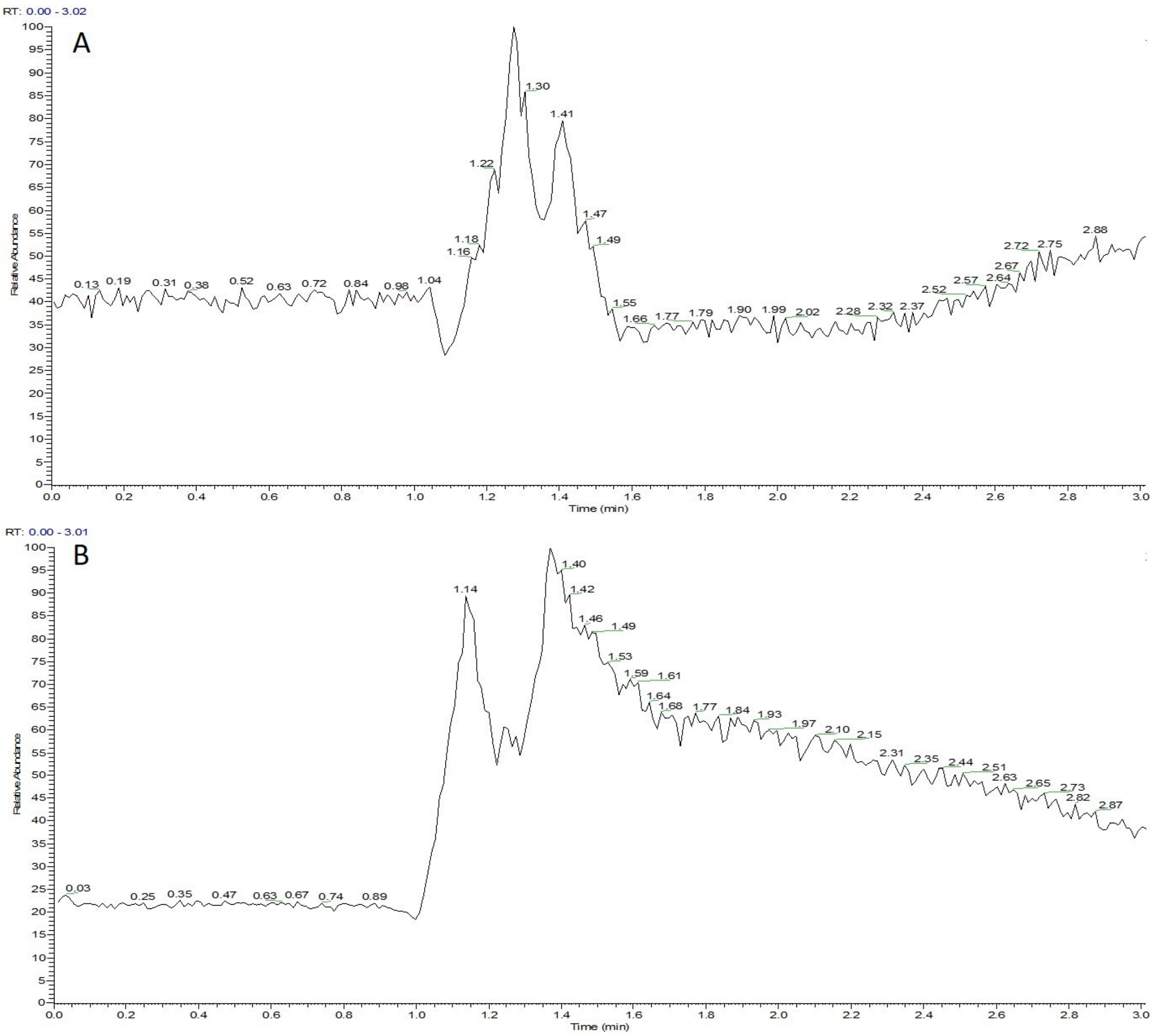

Supporting Figure 1. A three-minute LCMS method for high-throughput metabolomics. A: one representive chromatogram plot. The eluted metabolites were detected by a Q Exactive Plus mass spectrometer (Thermo Fisher) operated in full scan setup using electrospray positive mode. B: one representive chromatogram plot. The eluted metabolites were detected by a Q Exactive Plus mass spectrometer (Thermo Fisher) operated in full scan setup using electrospray negative mode.

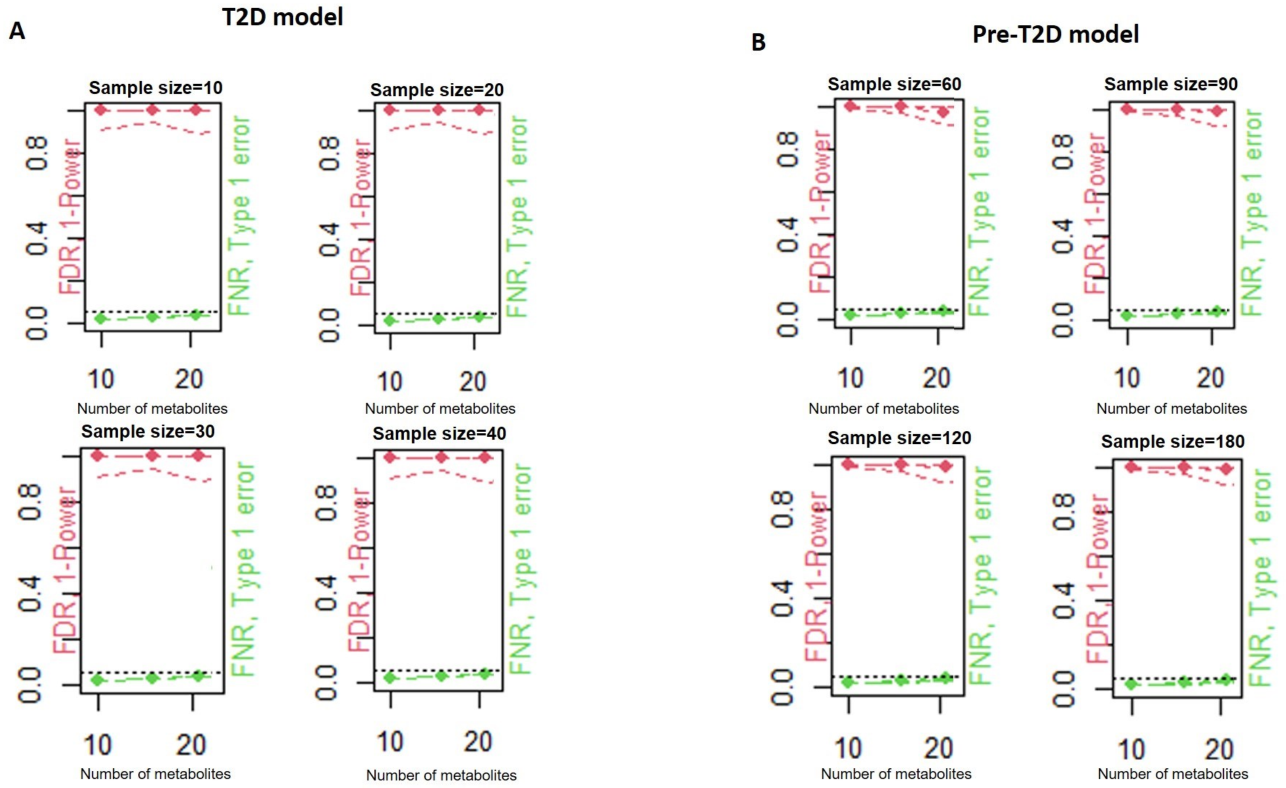

Supplemental Figure 2. Results for power analysis in (A) T2D classification and (B) Pre-T2D classification model. Each panel shows the estimated FDR and FNR (solid red and green curves) as well as the 10 and 90th percentiles. A horizontal line is drawn at 0.05. The quantity on the horizontal axis – number of predictive features – refers to both the hypothesized number of truly non-null features, and the number of features called significant. This analysis supports our testing sample cohort assembly with the sufficient sample size to test the predictive panel.
